# Immunogenic *Burkholderia pseudomallei* Outer Membrane Proteins as Potential Candidate Vaccine Targets

**DOI:** 10.1371/journal.pone.0006496

**Published:** 2009-08-05

**Authors:** Yuka Hara, Rahmah Mohamed, Sheila Nathan

**Affiliations:** 1 Malaysia Genome Institute, UKM-MTDC Smart Technology Centre, Bangi, Selangor, Malaysia; 2 School of Biosciences and Biotechnology, Faculty of Science and Technology, Universiti Kebangsaan Malaysia, Bangi, Selangor, Malaysia; Cairo University, Egypt

## Abstract

**Background:**

*Burkholderia pseudomallei* is the causative agent of melioidosis, a disease of significant morbidity and mortality in both human and animals in endemic areas. There is no vaccine towards the bacterium available in the market, and the efficacy of many of the bacterium's surface and secreted proteins are currently being evaluated as vaccine candidates.

**Methodology/Principal Findings:**

With the availability of the *B. pseudomallei* whole genome sequence, we undertook to identify genes encoding the known immunogenic outer membrane protein A (OmpA). Twelve OmpA domains were identified and ORFs containing these domains were fully annotated. Of the 12 ORFs, two of these OmpAs, Omp3 and Omp7, were successfully cloned, expressed as soluble protein and purified. Both proteins were recognised by antibodies in melioidosis patients' sera by Western blot analysis. Purified soluble fractions of Omp3 and Omp7 were assessed for their ability to protect BALB/c mice against *B. pseudomallei* infection. Mice were immunised with either Omp3 or Omp7, subsequently challenged with 1×10^6^ colony forming units (cfu) of *B. pseudomallei* via the intraperitoneal route, and examined daily for 21 days post-challenge. This pilot study has demonstrated that whilst all control unimmunised mice died by day 9 post-challenge, two mice (out of 4) from both immunised groups survived beyond 21 days post-infection.

**Conclusions/Significance:**

We have demonstrated that *B. pseudomallei* OmpA proteins are immunogenic in mice as well as melioidosis patients and should be further assessed as potential vaccine candidates against *B. pseudomallei* infection.

## Introduction

*Burkholderia pseudomallei* is a gram-negative facultative anaerobic motile bacillus that is the causative agent of melioidosis, an infectious disease resulting in significant morbidity and mortality for both humans and animals in endemic regions such as Southeast Asia and northern Australia [1], [2]. Melioidosis can present in many clinical forms, from acute pneumonia or septicaemia to chronic and subclinical forms and this poses a great challenge in rapid and accurate diagnosis of the disease [3]. Furthermore, therapy is complicated by antibiotic resistance in many clinical isolates, resulting in frequent relapse of patients and the mortality rate of patients with septic shock is approximately 80–95% despite treatment with ceftazideme, imipenem or meropenem [2]. Therefore, prevention, rather than cure of melioidosis, is critical.

Various vaccination strategies have been extensively explored. Recent work demonstrated significant protection in animal models following vaccination with attenuated strains of *B. pseudomallei* [4]. Attenuated strains for vaccination would require proof of avirulence as even *ara* ^+^ *B. thailandensis*, which is generally considered avirulent, can also cause clinical infection in humans [5], an important implication for vaccine research. Other approaches investigated include the use of dendritic cells as a delivery vector to generate cell-mediated immunity [6], conjugate vaccines of capsular polysaccharides and O-polysaccharide [7], [8] and the DNA vaccine against *fliC* flagellin structural gene [9].

With the advances in whole-genome sequencing and bioinformatics, one can use the genomic information to discover novel antigens which may have been missed by conventional methods. In this study, we adopted a bioinformatics-based approach to identify potential protective antigens in *B. pseudomallei* by using the genome information of *B. pseudomallei* K96243 made available by The Wellcome Trust Sanger Institute. We selected putative outer membrane protein A (OmpA) as OmpAs are often involved in bacterial virulence and immunity, have good immunogenic properties and are therefore, important vaccine candidates [10], [11]. Indeed, OmpAs from *Porphyromonas gingivalis*, a periodontal pathogen implicated in chronic periodontitis has been demonstrated to confer a high level of protection in the *P. gingivalis* murine lesion model [12]. In this study, we sought to determine the immunogenic properties of *B. pseudomallei* recombinant OmpAs and their ability to protect mice from *B. pseudomallei* infection. Our data demonstrate that two recombinant OmpAs evaluated were immunogenic in mice and show potential as candidate vaccine targets.

## Results

### Identification of putative *B. pseudomallei* OmpA genes

**Figure 1:**
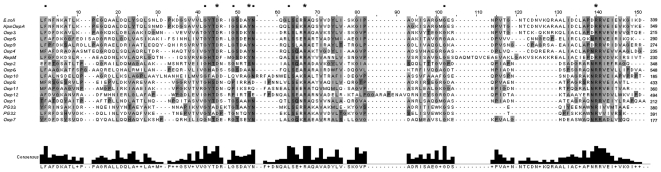
Multiple sequence alignment (C-termini) of predicted amino acid sequences of *B. pseudomallei* D286 OmpA. The predicted amino acid sequences of 12 *B. pseudomallei* OmpAs together with 5 experimentally verified immunogenic OmpAs were aligned. The experimentally verified OmpAs used for the alignment: *E. coli* OmpA (NP_415477.1), KpmOmpA - *K. pneumoniae* OmpA (ABR76422), PG33 - *P. gingivalis* OmpA (AF175715), PG32 - *P. gingivalis* OmpA (AF175714), RmpM–*N. meningitidis* OmpA (YP_001599860). The residues implicated for their role in the interactions with peptidoglycans are indicated (asterisks - direct interaction; dots - indirect interaction).

**Figure 2:**
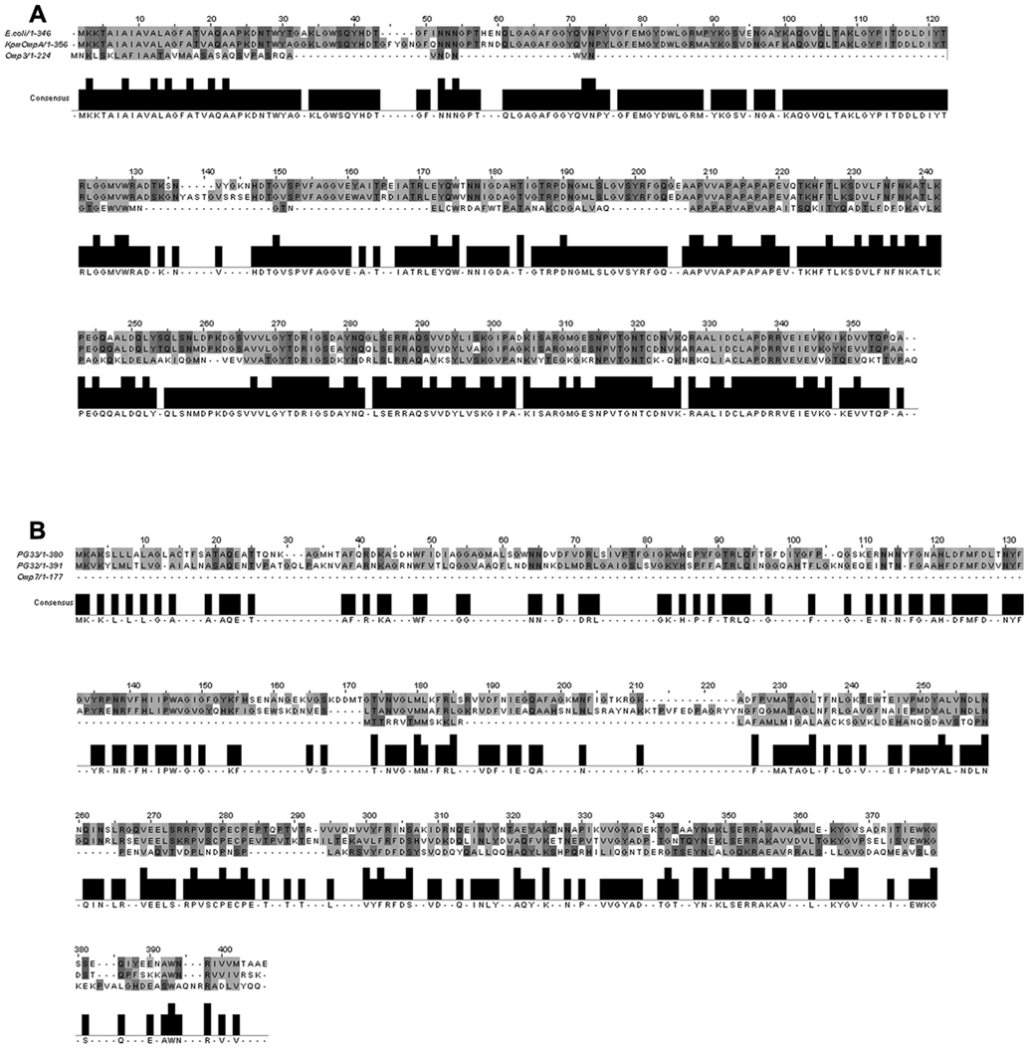
Multiple sequence alignment of Omp3 and Omp7 with known immunogenic OmpAs. Omp3 was aligned with *E. coli* OmpA (NP_415477.1) and *K. pneumoniae* OmpA (ABR76422) while Omp7 was aligned with *P. gingivalis* OmpAs PG33 (AF175715) and PG32 (AF175714).

We performed a BLASTP search using the conserved OmpA domain sequence (PF00691) as the query sequence and identified 14 open reading frames (ORFs) coding for proteins containing the OmpA conserved domain in the *B. pseudomallei* K96243 reference genome sequence. Of these 14 proteins, 13 hits had an E-value of 1e^−4^ or better, however, ORF 13 was not included for further studies as it was annotated as a putative cytochrome C oxidase (Table 1). Subsequently, 12 ORFs coding for OmpA-like proteins were selected for further analysis. Conservation of the OmpA domain sequence at the C-terminal of all 12 *B. pseudomallei* OmpA sequences as well as in 5 experimentally verified OmpAs [*Klebsiella pneumoniae* OmpA (ABR76422), *Escherichia coli* OmpA (NP_415477.1), *P. gingivalis* OmpA PG33 (AF175715), *P. gingivalis* OmpA PG32 (AF175714) and *Neisseria meningitidis* OmpA RmpM (YP_001599860)] is shown in Fig. 1. Residues previously proposed to be involved in *N. meningitidis* RmpM direct (D45, Y53, R67, and R140) and indirect (F2, G42, G49, N54, L63, G102) interactions with peptidoglycans are present in *B. pseudomallei* OmpA sequences and are denoted as asterisks and dots in Fig. 1. In addition, the multiple sequence alignment demonstrated that Omp3 clustered together with *E. coli* OmpA and *K. pneumoniae* OmpA while Omp7 clustered closely with *P. gingivalis* OmpAs. N-terminal sequences, on the other hand, were highly heterogeneous. Analysis of the global sequence similarity and calculated percentage identity demonstrated that both Omp3 and Omp7 revealed considerable similarity to reference proteins (Fig. 2). Omp3 was 40% identical to both *E. coli* and *K. pneumoniae* OmpA proteins while Omp7 was 21–23% identical to *P. gingivalis* OmpA proteins.

**Table 1:** Putative OmpA genes identified by BLASTP analysis using the OmpA domain sequence (below) as the query against the *B. pseudomallei* K96243 reference genome sequence.

Designation	Sanger annotation	BLAST score	Probability (N)
Omp1	putative OmpA family transmembrane protein	191	6.6e-17
Omp2	putative outer membrane protein	177	4.2e-15
Omp3	OmpA outer membrane protein precursor	211	5.0e-19
Omp4	OmpA family protein	171	8.7e-15
Omp5	putative exported protein	172	1.3e-14
Omp6	OmpA family membrane protein	173	5.2e-14
Omp7	putative OmpA family lipoprotein	161	1.0e-13
Omp8	OmpA family membrane protein	150	1.0e-11
Omp9	OmpA family protein	160	2.3e-13
Omp10	OmpA family membrane protein	125	8.8e-10
Omp11	OmpA family membrane protein	146	2.6e-11
Omp12	OmpA family membrane protein	88	7.0e-05
ORF13	putative cytochrome c oxidase	125	6.9e-09
ORF14	hypothetical protein	81	0.00029

Conserved OmpA domain sequence used as a query sequence (PF00691):

LFDFDKATLKPEQQQLLDAIADLLKAIPPDNRVIVEGHTDSRPIGSDEYPSNQALSERRADSVADYLVSKGGVPADRISAVGYGESKPIASNKTEEGRAKNR.

### Cloning, protein expression and purification of OmpA

Primers were designed to amplify the 12 OmpA-coding genes from the local clinical isolate, *B. pseudomallei* D286. The size of the predicted ORFs ranged from 513–1677 bp. The amplicons were successfully cloned into the pET101/D-TOPO expression vector for recombinant protein production. The full-length sequences of the inserts were verified by DNA sequencing (data not shown).

**Figure 3:**
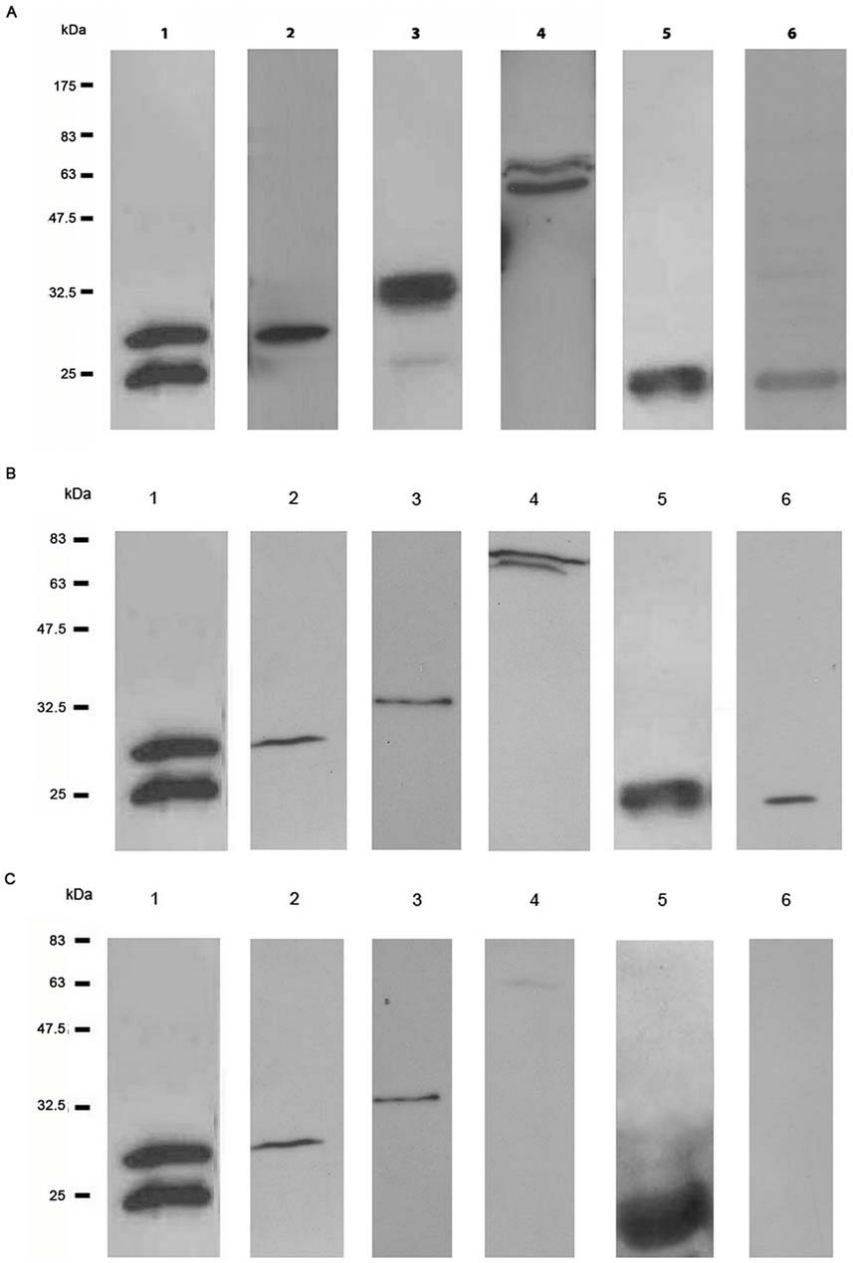
Western blot analysis of purified recombinant OmpA. Purified recombinant OmpAs were electrophoresed on a 12% SDS-PAGE, transferred onto a nitrocellulose membrane and reacted with (A) monoclonal anti-histidine tag antibody conjugated with HRP. The expected protein sizes for Omp3, 4, 5, 6, 7 and 10 are 27, 27.5, 34, 65, 22.5 and 24.5 kDa respectively. Molecular weight sizes (kDa) of the broad range marker are indicated in the left panel. Among 6 recombinant OmpA proteins, Omp3 and Omp7 reacted strongly with (B) rabbit anti-*B. pseudomallei* sera and (C) pooled melioidosis patients' sera compared to other Omps. Lane 1; Omp3, lane 2; Omp4, lane 3; Omp5, lane 4; Omp6, lane 5; Omp7, lane 6; Omp10.

Expression of 12 *B. pseudomallei* recombinant outer membrane proteins was carried out and various parameters such as induction time, isopropyl β-D-1-thiogalactopyranoside (IPTG) concentration and growth temperature, were optimized. Eight recombinant proteins were successfully expressed, of which 6 were soluble and 2 were insoluble proteins. We attempted to re-solubilise both insoluble recombinant proteins using high concentrations of the denaturants urea or guanidinium hydrochloride followed by dialysis. However, neither strategy resulted in sufficient amounts of soluble protein for subsequent analysis; thus, further studies on the OmpA proteins were limited to 6 soluble recombinant proteins. All 6 soluble fusion proteins were successfully detected with anti-His tag antibody by Western blot analysis (Fig. 3A). The proteins range in size from 22.5 kDa to 65 kDa corresponding to the predicted ORF. The recombinant proteins were reacted with rabbit antiserum raised against *B. pseudomallei* whole cells. All 6 recombinant proteins reacted with rabbit antiserum, but the degree of reactivity varied with the strongest reactivity observed with Omp3 and Omp7 (Fig. 3B). Subsequently, all 6 recombinant proteins were also reacted with melioidosis patients' sera. We pooled sera from five patients with different disease presentations in order to detect a spectrum of reactive antigens. Pooling takes advantage of the different immune responses presented by the hosts to the pathogen throughout the course of infection. Among the 6 recombinant proteins tested, Omp10 did not show any reactivity while Omp6 reacted relatively weakly. Both Omp3 and Omp7 demonstrated strong reactivity towards pooled patients' sera (Fig. 3C). None of the 6 recombinant proteins were recognised by antibodies from *Legionella* patients' sera (*B. pseudomallei*-negative) (data not shown). A distinct band with slightly lower molecular weight was also detected for recombinant Omp3 and Omp6. It is presently unknown if the multiple bands correspond to different isoforms of Omp3 and Omp6 or was a result of proteolytic breakdown.

### Antibody response and potential protective ability of recombinant OmpA

**Figure 4:**
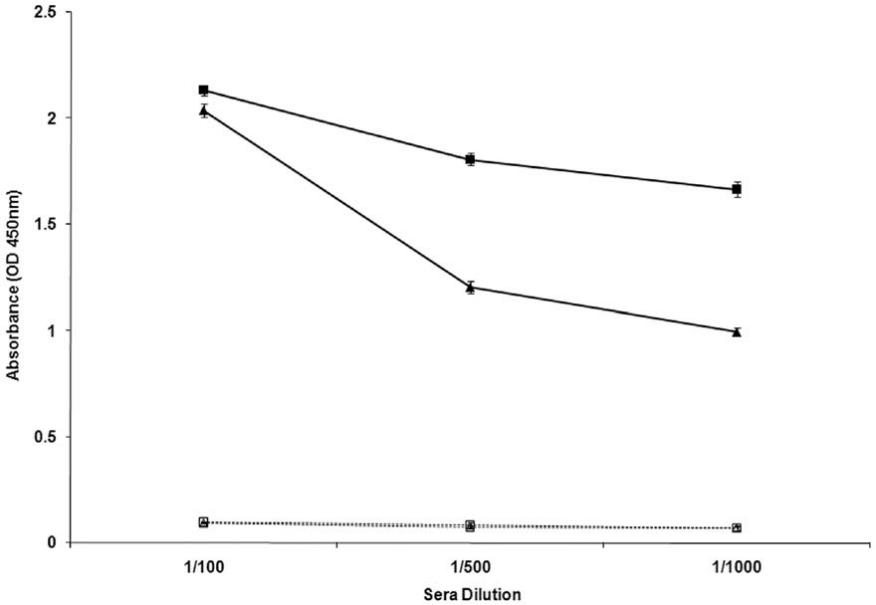
Screening of OmpA-specific IgG in immunised BALB/c mice. Mice were immunised with 50 µg of recombinant Omp3 or Omp7. The sera were collected (n = 4) prior to and after immunisation and pooled. The levels of OmpA-specific IgG were analysed by ELISA. The presence of specific antibodies to recombinant Omp3 (closed triangles) and Omp7 (closed squares) proteins was observed in sera from immunised mice but not in pre-immunised sera (open triangles for Omp3 pre-immunised sera and open squares for Omp7 pre-immunised sera) (*P*<0.05). Data represent the mean absorbance (OD_450nm_)±SEM.

Based on their strong reactivity towards anti-sera from *B. pseudomallei* infected humans and animals in addition to higher sequence similarity to known immunogenic OmpAs from other gram negative bacteria, Omp3 (Genbank accession number FJ746559) and Omp7 (Genbank accession number FJ746560) were selected to induce an antibody response and establish the ability of these two recombinant proteins to protect against *B. pseudomallei* infection in mice. To assess the immunogenicity of the recombinant Omp3 and Omp7, sera were collected and pooled from immunised mice prior to *B. pseudomallei* challenge, and tested for reactivity with purified OmpA by ELISA. The specific antibodies against recombinant Omp3 and Omp7 were observed in immunised mice with several dilutions screened, but, not in the pre-immunised sera (*P*<0.05) (Fig. 4). The absorbance value for anti-Omp7 antibodies was higher at all dilutions tested compared to anti-Omp3 antibodies, especially at higher dilutions.

**Figure 5:**
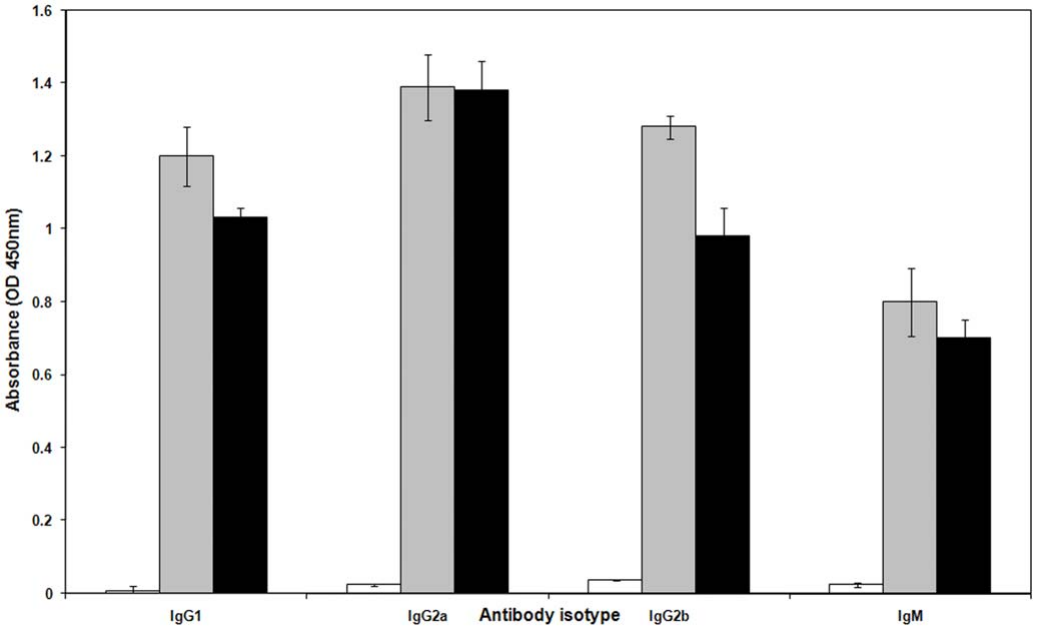
Analysis of OmpA-specific polyclonal antibody isotypes in immunised BALB/c mice. Mice were immunised with 50 µg of recombinant Omp3 or Omp7. The sera were collected (n = 4) prior to and after immunisation and pooled. The levels of IgG subclasses and IgM were determined by ELISA. Data represent the mean absorbance (OD_450nm_)±SEM. The IgG_2a_/IgG_1_ ratio of Omp3-immunised mice is 1.2 while for Omp7-immunised mice, the ratio is 1.3. Open bars represent pre-immunised sera, grey bars represent anti-Omp3 antibody and black bars represent anti-Omp7 antibody.

To identify the type of immune response induced, we determined specific IgG subclasses (IgG_1_, IgG_2a_, IgG_2b_) and IgM antibody titres by ELISA. The results indicated significantly higher titres (*P*<0.05) of IgG_1_, IgG_2a_, IgG_2b_ and IgM antibodies were produced in mice immunised with recombinant Omp3 and Omp7 when compared to antibody titres in the pre-immunised sera from the same mice. Immunisation with Omp3 induced higher antibody titres in all the antibody isotypes when compared to Omp7 immunised mice but the difference was not significant. The predominant Ig subclass elicited by Omp3 and Omp7 immunisation was IgG_2a_ followed by IgG_2b_ and to a lesser extent, IgG_1_. The ratio of IgG_2a_/IgG_1_ was 1.2 in Omp3 immunised mice and 1.3 in Omp7 immunised mice, respectively (Fig. 5).

From our previous work, the LD_50_ for the local clinical isolate used in this study, D286, was calculated to be 10^5^ cfu following infection of BALB/c mice via an intraperitoneal route. Similar LD_50_ values were previously reported for other *B. pseudomallei* clinical isolates [13] and we settled on a challenge dose of 10×LD_50_ as recommended by Ulett *et al*. [14]. The challenge dose of 10×LD_50_ is equivalent to LD_100_, whereby the dose should cause 100% death of the infected animals by the end of the observation period. Thus, any reduction in the mortality figures as a result of the immunisation would indicate the potential protective efficacy of these recombinant proteins.

**Figure 6:**
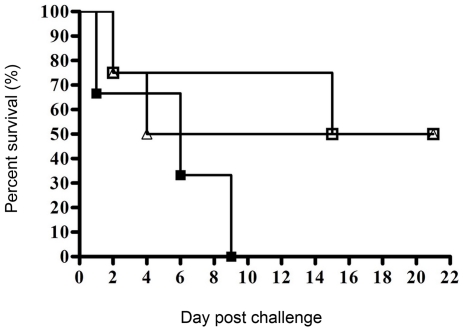
Survival of BALB/c mice immunised with recombinant Omp3 or Omp7 and challenged with *B. pseudomallei*. Mice were immunised with either Omp3 (open triangles) or Omp7 (open squares) prior to challenge with 1×10^6^ cfu *B. pseudomallei* by the i.p. route of infection. Both groups immunised with either Omp3 or Omp7 displayed 50% survival rate up to 21 days post-infection. All the control mice (closed squares) received *E. coli* BL21 (DE3) cells in FIA followed by challenge, and died by day 9 post-challenge.

For the control group of mice (n = 3) immunised with *E. coli* cells in adjuvant and subsequently challenged with *B. pseudomallei*, one mouse succumbed at 1-day post challenge, the second on day 6 and the last mouse died on day 9 with a median survival of 6 days (Fig. 6). For mice immunised with Omp3, one mouse (out of 4) died on day 2 and the second on day 4 (2/4) with a median survival of 13 days, while for mice immunised with Omp7, one mouse died on day 2 and the second on day 15 (2/4) with a median survival of 18 days. The remaining two mice from each group immunised with either Omp3 or Omp7 survived beyond 21 days post-challenge (Fig. 6). Immunisation with both recombinant proteins resulted in the prolonged median survival (7–12 days) in immunised animals compared to the control group.

In this pilot experiment, both recombinant Omp3 and Omp7 demonstrated protection in mice, but the degree of protection provided by immunisation using a single protein was incomplete (50%). The challenged survivors displayed significant splenomegaly and multiple abscess formation, demonstrating incomplete bacterial clearance from the system (data not shown).

## Discussion

*B. pseudomallei* is an intracellular pathogen and is known to survive and multiply within both phagocytic and non-phagocytic host cells and may be able to spread directly from cell to cell [15]. Thus, the nature of this bacterium highlights the need to generate cell-mediated immunity to combat infection. In an attempt to identify proteins capable of inducing a cell-mediated immune response, we selected putative *B. pseudomallei* proteins homologous to immunogenic OmpA to demonstrate proof-of-concept. We adopted a bioinformatics-based approach, and by utilising the reference genome sequence of *B. pseudomallei* K96243, we identified 12 putative OmpA genes in the genome. These genes contain the conserved OmpA domain at the C-termini (Fig. 1) while their N-terminal sequences share little similarity, another characteristic of OmpAs as they are generally heterogeneous at the N-terminal [16]. The C-terminal domain contains conserved residues previously proposed to be important for protein function and interaction with peptidoglycans in *N. meningitidis* [17]. Some residues may interact directly with peptidoglycans while others may have an indirect interact with peptidoglycans by stabilising residues involved in the direct interaction. All identified residues were well conserved in *B. pseudomallei* OmpAs, suggesting that they may play similar roles in *B. pseudomallei* OmpA interaction with peptidoglycans for maintenance of membrane and cell morphology.

OmpAs are known to be involved as virulence factors in bacterial pathogenesis and are capable of inducing both humoral and cytotoxic responses [11], [18]. OmpAs were previously proposed in the design of vaccines for bacterial infections caused by Gram negative bacteria such as *Vibrio cholerae* [10], *K. pneumoniae* [19] and *P. gingivalis* [12] and promising data from animal models have been reported. Thus, we expressed 6 soluble recombinant *B. pseudomallei* OmpAs to assess their immunogenicity and evaluate the potential protective efficacy in an animal model of melioidosis. The observed presence of antibodies towards this class of membrane protein in both the melioidosis animal model as well as human patients implicates a functional role for OmpA during active *B. pseudomallei* infection. Indeed, evidence indicates that OmpA is a determinant of bacterial virulence and that it is a major target of the mammalian host cell defence [20], [21]. The differences in immunoreactivity demonstrated by *B. pseudomallei* OmpAs may somewhat correlate to the sequence similarity of these OmpAs to experimentally verified immunogenic OmpAs. The strongly immunoreactive Omp3 and Omp7 were clustered together with known immunogenic OmpAs (from *E. coli, K. pneumonia* and *P. gingivalis*) whereas weakly reactive proteins (Omp6 and Omp10) were not as closely related. The percentage of sequence identity observed between Omp3/Omp7 and the reference proteins was moderate and predominantly at the C-terminal domain, however, they showed considerable global sequence similarity, and thus we speculate that they may be similar at the structural level when they are exposed to the host immune system. Furthermore, conservation of the C-terminal domain sequence is important since the C-terminal domain of *E. coli* OmpA is known to contain the immunodominant epitope and is efficiently recognised by antibodies formed during the course of infection [22]. Similar immunodominant epitope(s) may be better conserved in Omp3 and Omp7 than in the other *B. pseudomallei* OmpAs and/or exposed more efficiently, leading to better recognition by antibodies from infected individuals.

Vaccine development for melioidosis is made difficult because *B. pseudomallei* is an intracellular pathogen that can successfully evade the host immune system for an extended period of time. Effective vaccination against this pathogen is therefore required to elicit a cytokine-mediated cellular response so as to combat potential disease recurrence [23]. Various approaches have been undertaken towards the development of effective vaccines against *B. pseudomallei* infection in animal models. Those investigated include heat-killed or live attenuated mutants of *B. pseudomallei*, polysaccharides and a DNA-vaccine coding for the *fliC* flagellin structural gene [4], [7]–[9]. These studies demonstrated a significant protection against *B. pseudomallei* infection in immunised animal models, however, the level of protection varied depending on the different vaccine strategies. For example, immunisation with attenuated mutants of *B. pseudomallei* resulted in 60% survival rate at 35 days post challenge in immunised mice [4] while DNA-vaccination protected 85% of vaccinated mice up to 7 days post infection [9] but the observation period was limited. Active immunisation with *B. pseudomallei* lipopolysaccharide resulted in 50% survival at day 35 post challenge in immunised mice [8]. Thus, data from the present pilot study is generally comparable to these previously reported efficacies whereby immunisation with recombinant Omp3 or Omp7 could be seen to be as effective as immunisation with lipopolysaccharide in murine models of melioidosis. Furthermore, our assessment of polyclonal antibody isotypes in the sera from Omp3- and Omp7-immunised mice indicated a predisposition towards a T_h_1-driven immune response as demonstrated by an IgG_2a_/IgG_1_ ratio of >1 [24]. Both recombinant proteins demonstrated similar potential protective efficacy, however, Omp7 appeared to be more efficient in inducing a higher titre of anti-Omp7 antibodies and higher IgG_2a_/IgG_1_ ratio. This may have contributed to the improvement of the median survival in Omp7-immunised mice when compared to Omp3-immunised mice. However, challenged survivors were unable to clear the infection and a chronic infectious status was evidently present, whereby mice eventually succumbed to the infection. Such findings are consistent with previously reported studies that immunisation strategies alone are not sufficient to protect animals from infection. Furthermore, recombinant antigens as vaccination candidates typically induce strong humoral antibodies but may be hampered in their ability to induce the cellular-mediated immune response [25]. Eradication of *B. pseudomallei* has been proposed to be dependent on the induction of a T_h_1 type immune response at the onset of melioidosis [6]. This is because in the presence of protective humoral antibodies, *B. pseudomallei* is still able to invade epithelial or phagocytic cells, resulting in a long term intracellular latent infection in the infected individuals before relapse occurs many years later [26], [27].

The use of vaccine delivery vectors such as dendritic cells (DCs) has been proposed to be effective in inducing significant levels of protective immune response against heterologous strains of *B. pseudomallei* by eliciting strong cell-mediated immune responses and lower but adequate levels of antibody responses [28]. Although the use of cultured DCs as a human vaccine candidate is simply not viable, a vaccine strategy that actively targets DCs is feasible by means of a formulation carrying recombinant *B. pseudomallei* antigens and Toll-like receptor (TLR) ligands. *B. pseudomallei* binding to its known receptor, TLR2, would activate the DCs, initiating the process of antigen uptake, processing and presentation required for the generation of protective immune responses. Recombinant OmpAs from this study could be good candidate antigens for coupling to DCs.

In summary, this study has demonstrated that by using publicly available bacterial genome sequence information, we successfully identified two immunogenic antigens, Omp3 and Omp7. Although more extensive studies are required, data from this pilot experiment have demonstrated the promising protective efficacy of *B. pseudomallei* OmpAs against an animal model of melioidosis.

## Materials and Methods

### Ethics statement

All animal experiments were performed in accordance with the animal ethics guideline compiled by Universiti Kebangsaan Malaysia Animal Ethics Committee (UKMAEC). Animals were maintained under specific-pathogen-free conditions and on a 12-h light/dark cycle. New Zealand White rabbits and BALB/c mice were obtained from the Universiti Kebangsaan Malaysia Animal House Facility. The rabbits were housed in individual cages and had free access to food and water during the study. The mice were provided with pelleted protein-enriched diet and water *ad libitum*.

### In silico genome analysis and multiple sequence alignment

The conserved OmpA domain sequence PF00691 (Table 1) was used as a query sequence to perform BLASTP [29] analysis against the annotated genome sequence of *B. pseudomallei* clinical strain K96243 deposited at http://www.sanger.ac.uk/cgi-bin/blast/submitblast/b_pseudomallei. The sequences containing the conserved OmpA domain were selected using a cut-off threshold of 1e^−4^. Subsequently, full ORFs of these genes were determined using Artemis [30]. Multiple sequence alignment of *B. pseudomallei* OmpA sequences together with the 5 experimentally verified OmpAs from other gram negative bacteria [*K. pneumoniae* OmpA (ABR76422), *E. coli* OmpA (NP_415477.1), *P. gingivalis* OmpA PG33 (AF175715), *P. gingivalis* OmpA PG32 (AF175714) and *N. meningitidis* OmpA RmpM (YP_001599860)] was performed using ClustalW [31]. The percentage identity between the predicted amino acid sequences of *B. pseudomallei* OmpAs and immunogenic OmpAs from other bacteria was calculated using Jalview [32].

### Bacterial strain and preparation of genomic DNA

The *B. pseudomallei* strain D286 was previously isolated from a melioidosis patient at the Kuala Lumpur Hospital [33]. A stock culture of this strain was obtained from the Pathogen Laboratory, Faculty of Science and Technology, Universiti Kebangsaan Malaysia. Genomic DNA was extracted according to the standard protocol [34] with modification. Briefly, *B. pseudomallei* strain D286 was grown at 37°C in Brain Heart Infusion broth (Pronadisa, Spain) and then plated on Ashdown selective agar plates. A single colony was inoculated into fresh Brain Heart Infusion broth and grown overnight at 37°C, following which the bacterial cells were harvested and the cell pellet was dissolved in TE buffer (1 mM Tris-Cl, pH 7.5, 1 mM EDTA). The pellet was treated with Proteinase K at a final concentration of 25 µg/ml and 10% SDS (Amresco, USA) and incubated at 37°C for one hour. CTAB-NaCl (10% CTAB (Sigma, USA), 0.7 M NaCl) was added and the incubation was continued at 65°C for 20 min. Purification of genomic DNA was carried out according to the standard phenol chloroform method and precipitated using 0.6 V of isopropanol.

### Amplification of *B. pseudomallei* OmpA genes and cloning

Primer sequences used for amplification of the twelve ORFs from the *B. pseudomallei* strain D286 genome were designed based on the 12 annotated ORFs and are listed in Table 2. The forward primers contained an additional CACC sequence at the 5′-end to allow direct cloning into the expression vector. PCR was conducted in a final volume of 50 µl that contained 5 µl 10× Expand High Fidelity reaction buffer with 15 mM MgCl_2_ (Roche, Germany), 1 µl *B. pseudomallei* genomic template, 1 µl deoxynucleotide mix (10 mM each of dNTP) (Promega, USA), 1 µl each of forward and reverse primers (25 pmoles each), 5 U of Expand High Fidelity enzyme mix (Roche, Germany) and 40.25 µl of MilliQ water. Amplification was performed under the following conditions; 1 cycle of 95°C for 5 min; 10 cycles of 94°C for 15 s, 55°C for 30 s, and 72°C for 1 min; 20 cycles of 94°C for 15 s, 55°C for 30 s and 72°C for 1 min with 5 s cycle elongation for each successive cycle for the final step; and 1 cycle of 72°C for 7 min. To achieve high efficiency and regulation for cloning and expression in an *E. coli* expression system, PCR products were cloned into pET101/D-TOPO® according to the manufacturer's recommendations (Invitrogen, USA). The gene orientation and sequence fidelity was verified by DNA sequencing.

**Table 2:** Primer sequences used for amplification of the 12 predicted ORFs of *Burkholderia pseudomallei* OmpA.

Sanger ID	Designation	Forward sequence	Reverse sequence
BPSL0999	Omp1	>5′caccatgaataccaaaa	<5′ctgcgccgcttgc
BPSL3099	Omp2	>5′caccatgcgcaaaacgac	<5′ctgtcccgcgcggaattc
BPSL2522	Omp3	>5′caccatgaataaactttcaaagctc	<5′ctgcgccggaacggtcgtc
BPSL2062	Omp4	>5′caccatgttcaagac	<5′cgctccgtcgagccccgc
BPSL1659	Omp5	>5′caccatgaattctactaacg	<5′cttttgcttgatgcggatttc
BPSS0096	Omp6	>5′caccatgtcgcgtcagattc	<5′tgatcccgtaccgctcgccttcctg
BPSL2765	Omp7	>5′caccatgaccacaaggag	<5′ctgttgatagacgagg
BPSS0102	Omp8	>5′caccatgaattcgggc	<5′cagcgtgaccgcgatttcga
BPSS0909	Omp9	>5′caccatgaacaggatgtcgaaata	>5′gcgccgttcgccggcgacgtc
BPSS1264	Omp10	>5′caccatgtcggtgctgctc	<5′tccacgacgctcaccg
BPSS0168	Omp11	>5′caccatgcccgagcgg	<5′gcgcgcgaccgtgatttc
BPSS0531	Omp12	>5′caccatgctcgatcgcg	<5′tggctgacggccctccgtggtcgg

### Recombinant OmpA protein production and purification

Transformation of host BL21 Star (DE3) cells was performed according to the manufacturer's recommendations (Invitrogen, USA). The positive control used for expression was *E. coli* BL21 Star (DE3) transformed with pET-101-LacZ (Invitrogen, USA). The colonies were cultured overnight and used to inoculate fresh LB medium supplemented with carbenicillin (50 µg/ml) (Phytotechnology Laboratories, USA). At OD_600nm_ = 0.5–0.6, expression was induced with 1 mM IPTG (Promega, USA) for 4 hr at 30°C. At the end of protein induction, cells were harvested and all protein extraction procedures were performed at 4°C. The cell pellet was resuspended in l ml lysis buffer (50 mM NaH_2_PO_4_, 300 mM NaCl, 10 mM imidazole, pH 8.0) and stored at −20°C overnight. The cells were then sonicated, centrifuged and the supernatant containing the soluble proteins was collected. His6-tagged recombinant protein purification was performed with the Ni-NTA purification system (QIAgen, USA). The column was washed with wash buffer (50 mM NaH_2_PO_4_, 300 mM NaCl, 20 mM imidazole, pH 8.0) and then protein fractions were eluted with elution buffer (50 mM NaH_2_PO_4_, 300 mM NaCl, 250 mM imidazole, pH 8.0). The eluted protein fractions were concentrated by centrifugation (Vivaspin, MW cutoff 10 kDa) (Vivascience, Germany) and protein concentration was determined using the bicinchoninic acid assay (BCA) (Sigma, USA) [35].

### Electrophoresis and Western blotting

Sodium dodecyl sulphate polyacrylamide gel electrophoresis (SDS-PAGE) was carried out according to Laemmli [36] using a 12% polyacrylamide gel. Protein samples (30 µg) were separated under reducing conditions and subsequently transferred to a nitrocellulose membrane (Amersham, USA) [37] and subjected to Western blotting with anti-His (C-term) HRP antibody (Invitrogen, USA) (dilution 1∶3000) and detected using SuperSignal® West Pico chemiluminescent HRP substrate (Pierce, USA). Recombinant proteins were also probed with rabbit anti-*B. pseudomallei* sera (see below) (dilution 1∶5000), pooled melioidosis patients' sera (dilution 1∶1600) or pooled patients' sera with *Legionella* infection (*B. pseudomallei*-negative) followed by goat anti-rabbit IgG conjugated with HRP (Promega, USA) (dilution 1∶10,000) or anti-human antibody IgG with HRP (Sigma, USA) (dilution 1∶4000) respectively, and signals were detected as above.

### Human sera samples

Culture-confirmed melioidosis sera of five adult patients from Malaysia presenting with acute and chronic disease manifestations were pooled and used. The serum samples from two patients presenting with *Legionella* infection but confirmed *B. pseudomallei*-negative were pooled and used as the control. A series of dilutions was performed on the serum samples to determine the optimal titres for Western analysis. All the serum samples were generously provided by the Institute for Medical Research, Malaysia.

### Rabbit anti-*B. pseudomallei* serum

Rabbit anti-serum was raised against heat killed (80°C, 1 h) whole *B. pseudomallei* cells. Heat-treated cells were injected into two male New Zealand White rabbits. The first rabbit received 5×10^5^ cfu in Freund's incomplete adjuvant (FIA) while the second rabbit received 2.5×10^5^ cfu in FIA, respectively, for 2-week intervals over 2 doses. Rabbits were bled 2 weeks after the second dose and sera samples were collected. ELISA was performed according to the protocol described elsewhere [38] using heat-killed whole *B. pseudomallei* cells to test the sera reactivity and calculate the optimal titre for immunoblotting.

### Antibody analysis and mouse protection study

BALB/c mice (n = 4, 6 weeks, female) were immunised with three doses of 50 µg purified recombinant Omp3 or Omp7, via the intraperitoneal route (i.p.) over 9 weeks. The first dose was administered in Freund's complete adjuvant (FCA) while the latter two doses were in FIA, three weeks apart. For antibody analysis, the sera samples were collected (n = 4) prior to immunisation one week after the second booster and pooled. The antibody titre of total IgG was analysed by ELISA as previously described [38]. Briefly, the 96-well microplates were coated with 0.5 µg per well of either purified recombinant Omp3 or Omp7, or crude *E. coli* protein in coating buffer (0.1 M sodium bicarbonate, p.H 8.6) and incubated overnight at 4°C. The plates were blocked, washed, then incubated with serial dilutions of sera samples and incubated at 37°C for 1 h. The diluted Rabbit anti-mouse IgG conjugated with peroxidase (1∶4000, Pierce, USA) was added and incubation continued for 1 h at 37°C. At the end of the incubation, the plates were washed and 1-step™ Ultra TMB-ELISA (Pierce, USA) was added as substrate. The reaction solutions were read at 450 nm using a microplate reader.

For detection of Omp3 and Omp7 polyclonal antibody isotypes, plates coated with recombinant Omps were probed with mouse sera as primary antibodies followed by the addition of specific anti-mouse IgG_1_-, IgG_2a_-, IgG_2b_- or IgM-HRP conjugated secondary antibodies (1∶1000 for IgG, and 1∶2000 for IgM) (Zymed, CA, USA).

The LD_50_ of the *B. pseudomallei* local isolate D286 via an i.p. route of infection was previously calculated at approximately 1×10^5^ cfu in a BALB/c mouse model (data not shown). Thus, 1×10^6^ cfu (10×LD_50_) were used in this study as the lethal dose for infection. Mice were challenged intraperitoneally 2 weeks after the second booster with approximately 1×10^6^ cfu *B. pseudomallei* D286. Mice were examined daily for 21 days post-challenge. Control mice (n = 3) received *E. coli* BL21 (DE3) cells in FIA followed by challenge.

### Statistical analysis

Statistical analysis on anti-OmpA mice sera reactivity was performed using the Mann Whitney test within the SPSS (version 14.0) software package. Data are expressed as the mean±SEM. Survival (%) was calculated using the Kaplan-Meier Cumulative Survival Plot for Time (nonparametric survival analysis) and survival curves were compared using Log Rank tests (GraphPad Prism) where *P* values of <0.05 were taken to be significant. All experiments were replicated in a comparable fashion and found to produce findings similar to the data presented. The median survival is defined as the time at which half the subjects have died.
